# Supplementary figures and images for: Integrated epidemiologic investigation and genomic confirmation of a Klebsiella pneumoniae neonatal sepsis outbreak in Botswana
Source: PLOS Glob Public Health. 2026 Jun 15;6(6):e0006468. doi: 10.1371/journal.pgph.0006468 (PMC13268195; doi:10.1371/journal.pgph.0006468)

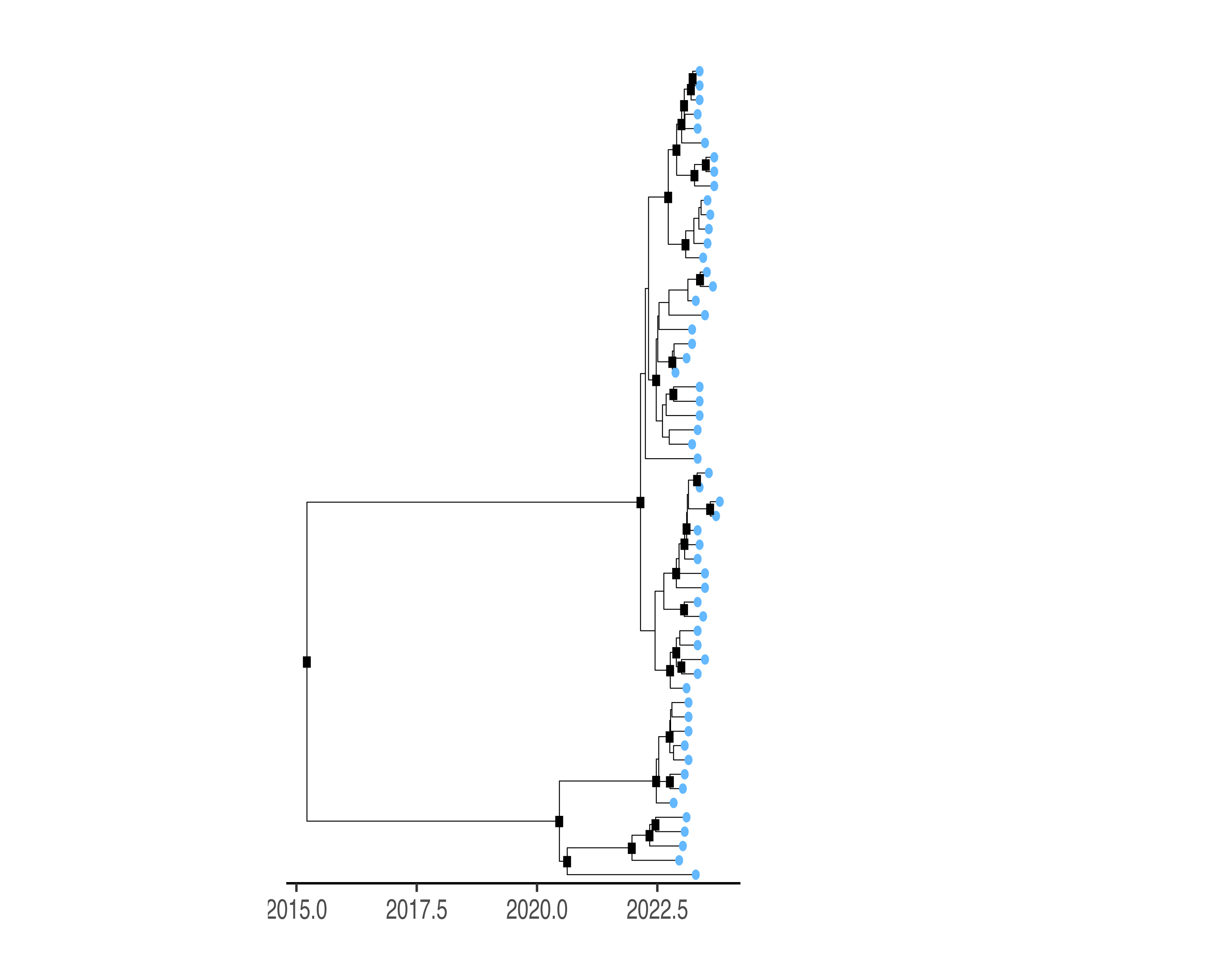

Supplement: S1 Fig — Blue tip color indicates colonization isolate, and posterior branch supports more than 0.75 are indicated with black boxes. Isolates were identified from colonization screening (skin or perirectal) for multidrug-resistant Kpn collected during surveillance; no detections from environmental or bloodstream infection isolates during this period. (TIF) [file pgph.0006468.s003.tif]

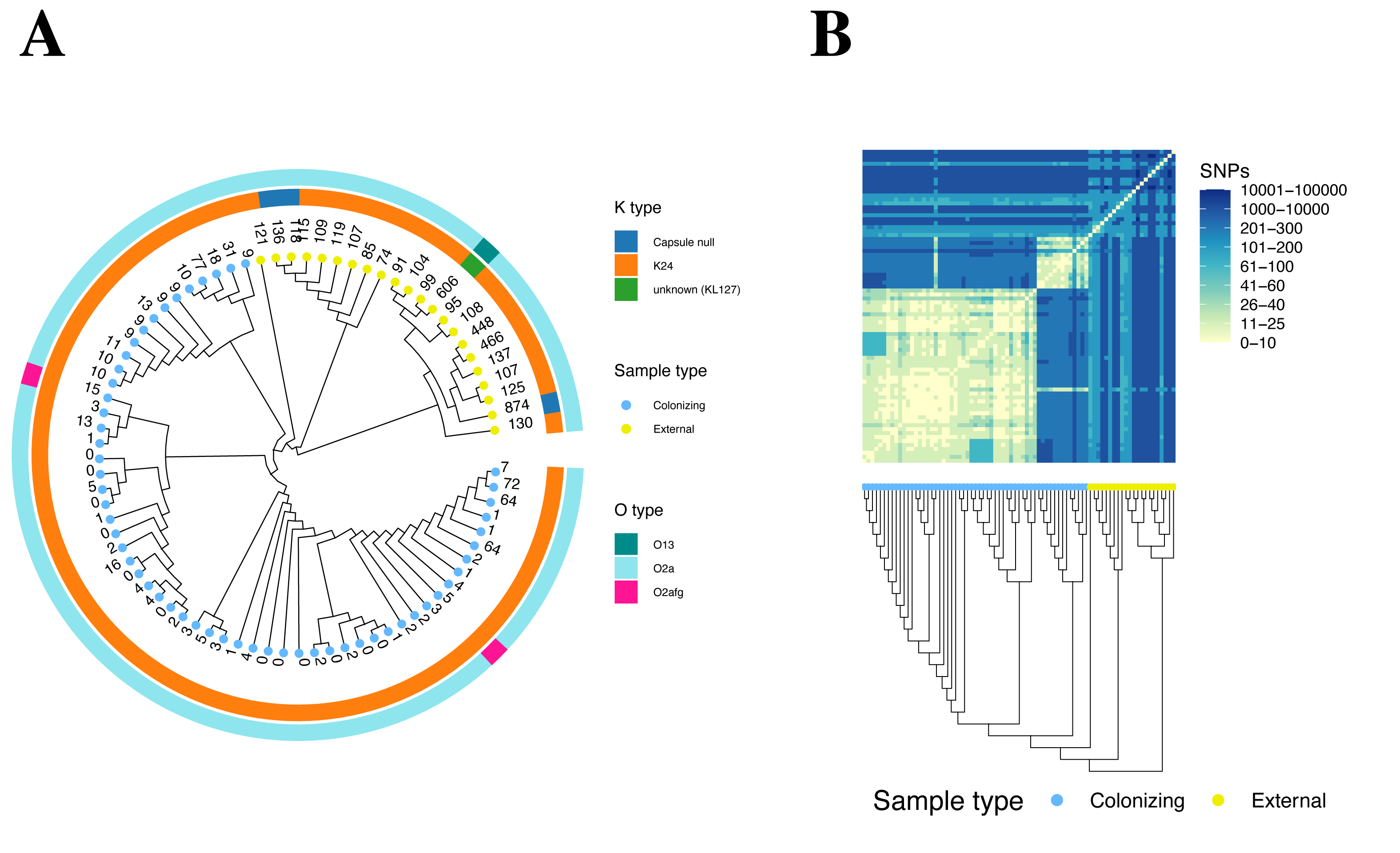

Supplement: S2 Fig — B. A heatmap produced using SNP-dist. Closest public genomes from NCBI were identified using WhatsGNU and were included. Blue and yellow tip color indicate study colonization isolates and external public genomes, respectively. (TIF) [file pgph.0006468.s004.tif]

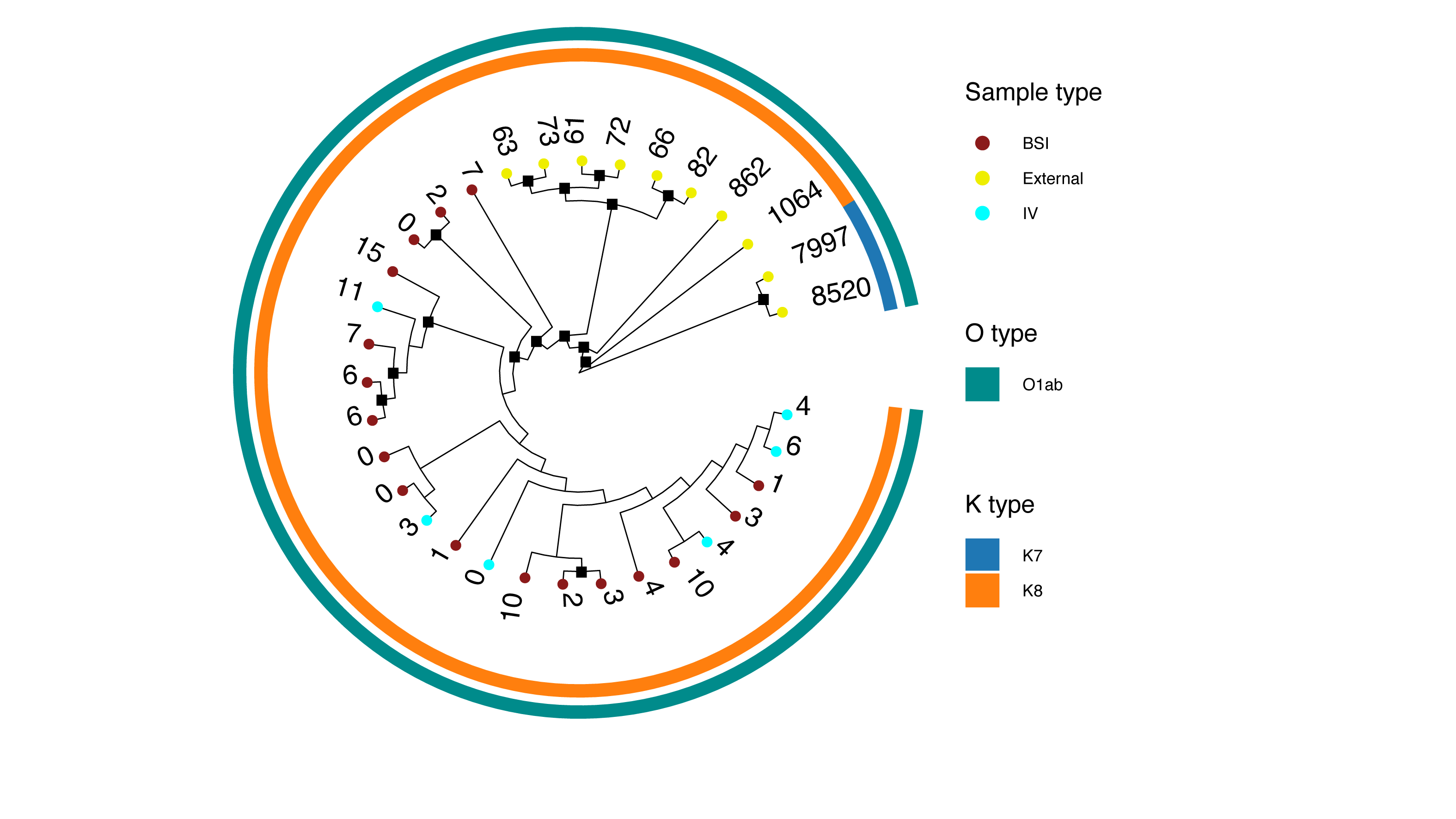

Supplement: S3 Fig — NNU.28.dextrose.dup within the ST using Snippy. Closest public genomes from NCBI were identified using WhatsGNU and were included. Bootstrap values above 75 are shown on the branches as black squares. Numbers on the tips represent the SNP distance from the reference. Red, cyan and yellow tip colors indicate study bloodstream infection isolates, intravenous fluid isolates, and external public genomes, respectively. (TIF) [file pgph.0006468.s005.tif]

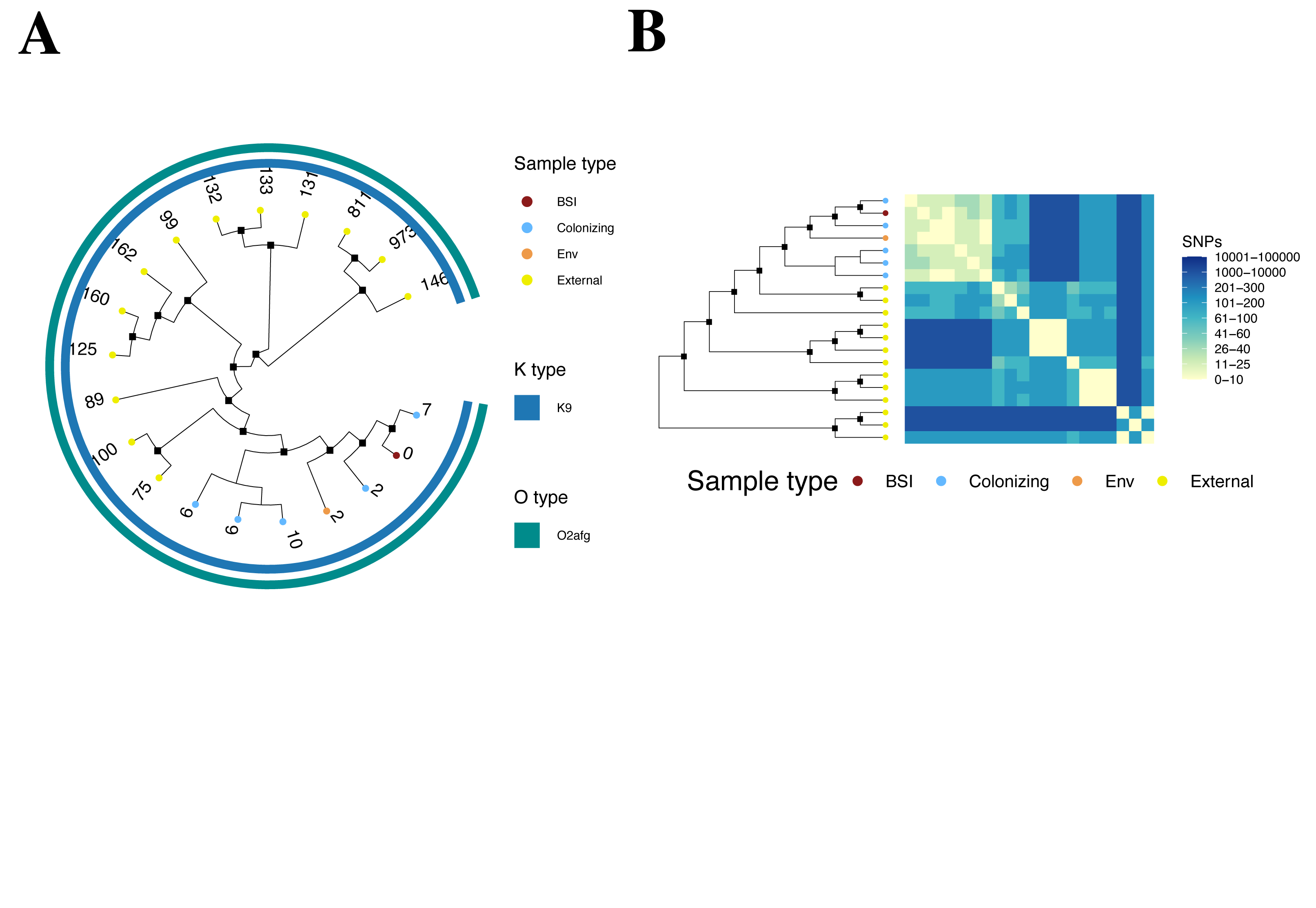

Supplement: S4 Fig — B. A heatmap produced using SNP-dist. Closest external public genomes from NCBI were identified using WhatsGNU and were included. Bootstrap values above 75 are shown on the branches as black squares. Numbers on the tips represent the SNP distance from the reference. Blue, red, orange, and yellow tip colors indicate study colonization isolates, bloodstream infection isolates, environmental isolates, and external public genomes, respectively. (TIF) [file pgph.0006468.s006.tif]

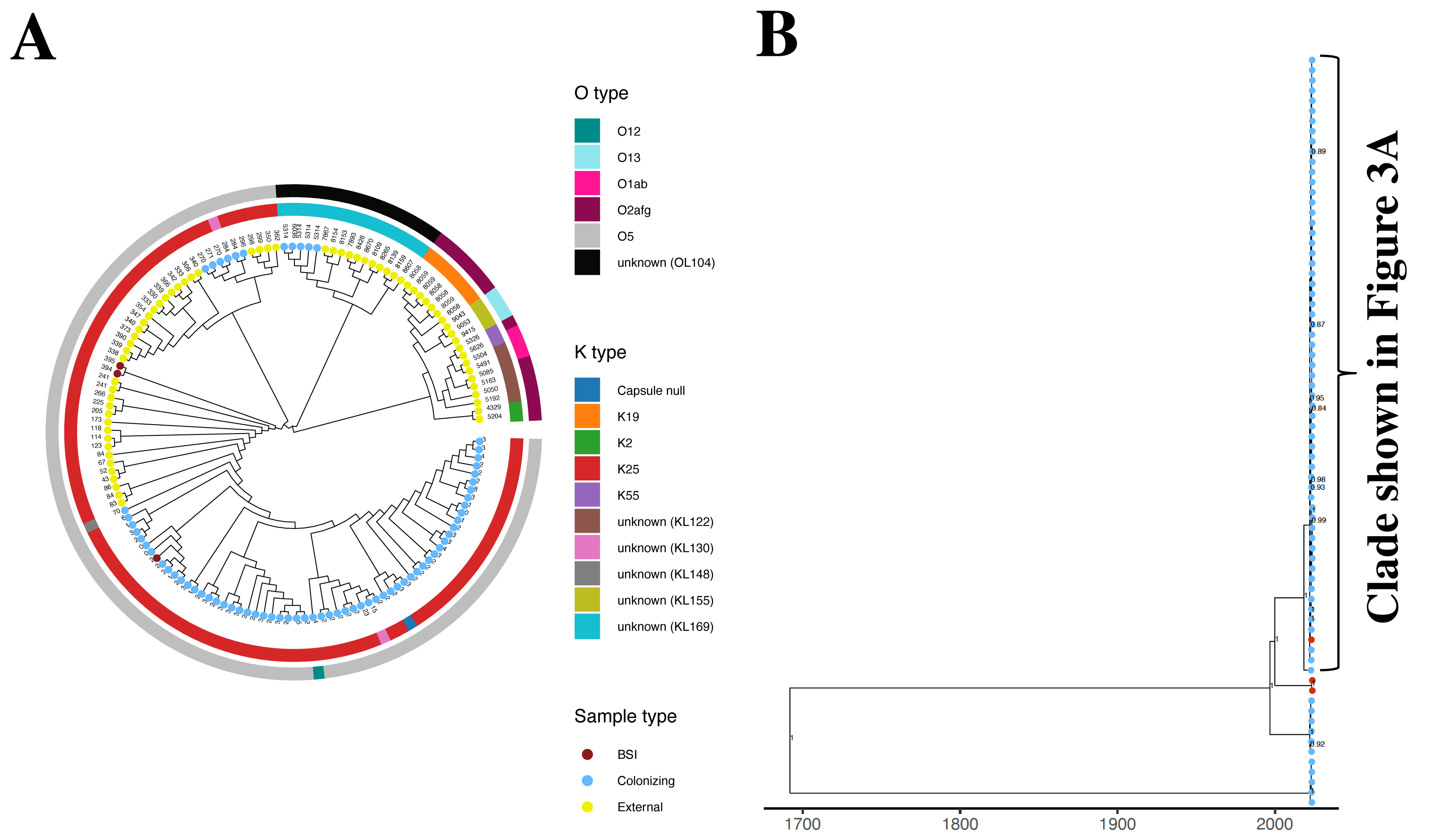

Supplement: S5 Fig — Closest public genomes from NCBI were identified using WhatsGNU and were included. Bootstrap values above 70 are shown on the branches. Numbers on the tips represent the SNP distance from the reference. Blue, red and yellow tip color indicate colonization, bloodstream (BSI) isolates, and external public genomes, respectively. B. Time-scaled BEAST phylogenetic tree of Klebsiella pneumoniae ST17. Blue, red, and yellow tip colors indicate colonization isolates, BSI isolates, and external public genomes respectively. Posterior branch supports above 0.75 are indicated where relevant. (TIF) [file pgph.0006468.s007.tif]

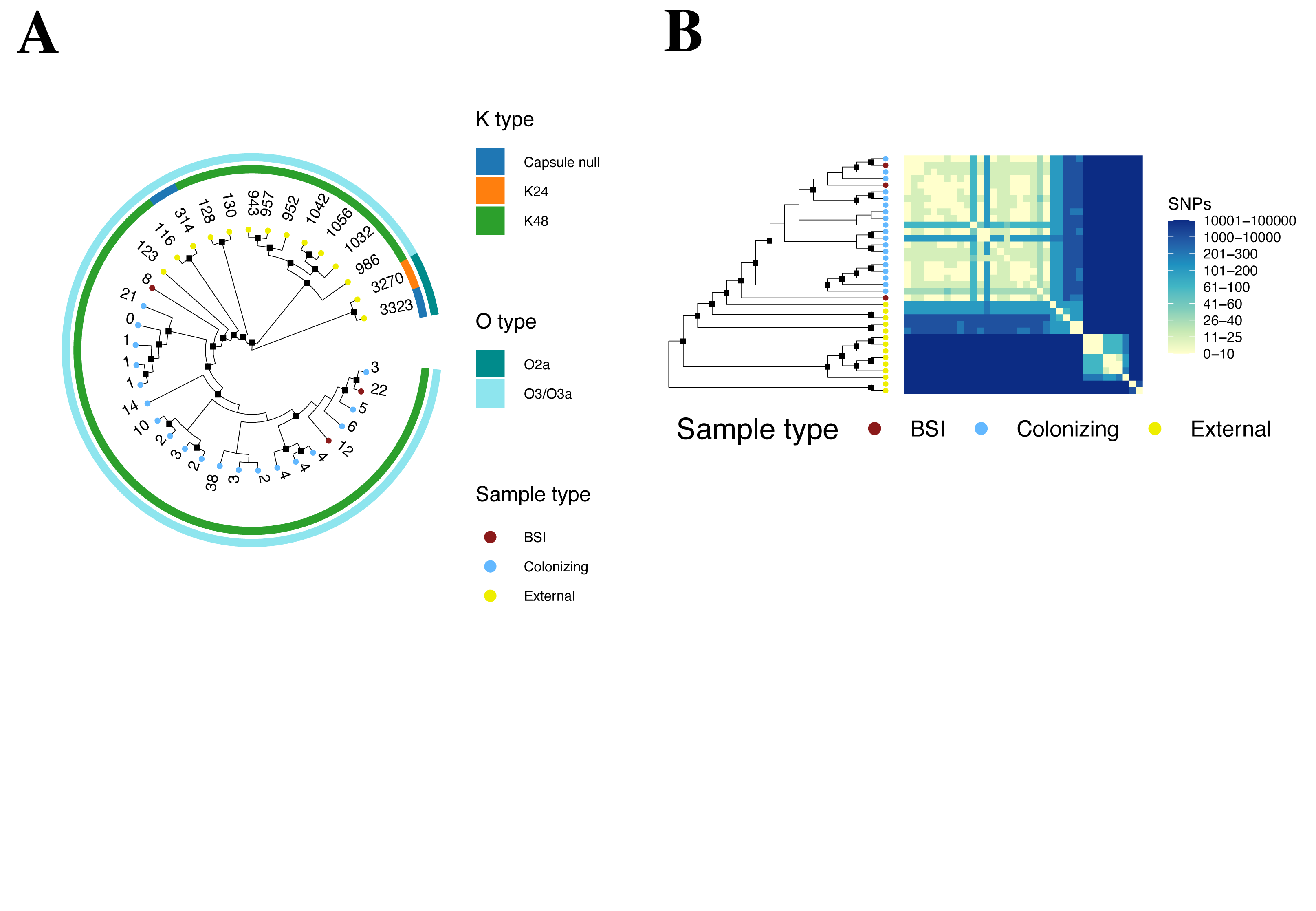

Supplement: S6 Fig — Numbers on the tips represent the SNP distance from the reference. Blue, red and yellow tip color indicate colonization, bloodstream (BSI) isolates, and external public genomes, respectively. B. a heatmap produced using SNP-dist. Closest public genomes from NCBI were identified using WhatsGNU and were included. Bootstrap values above 75 are shown on the branches as black squares. Numbers on the tips represent the SNP distance from the reference. Blue, red, and yellow tip colors indicate colonization isolates, BSI isolates, and external public genomes respectively. Bootstrap values above 75 are shown on the branches as black squares. (TIF) [file pgph.0006468.s008.tif]

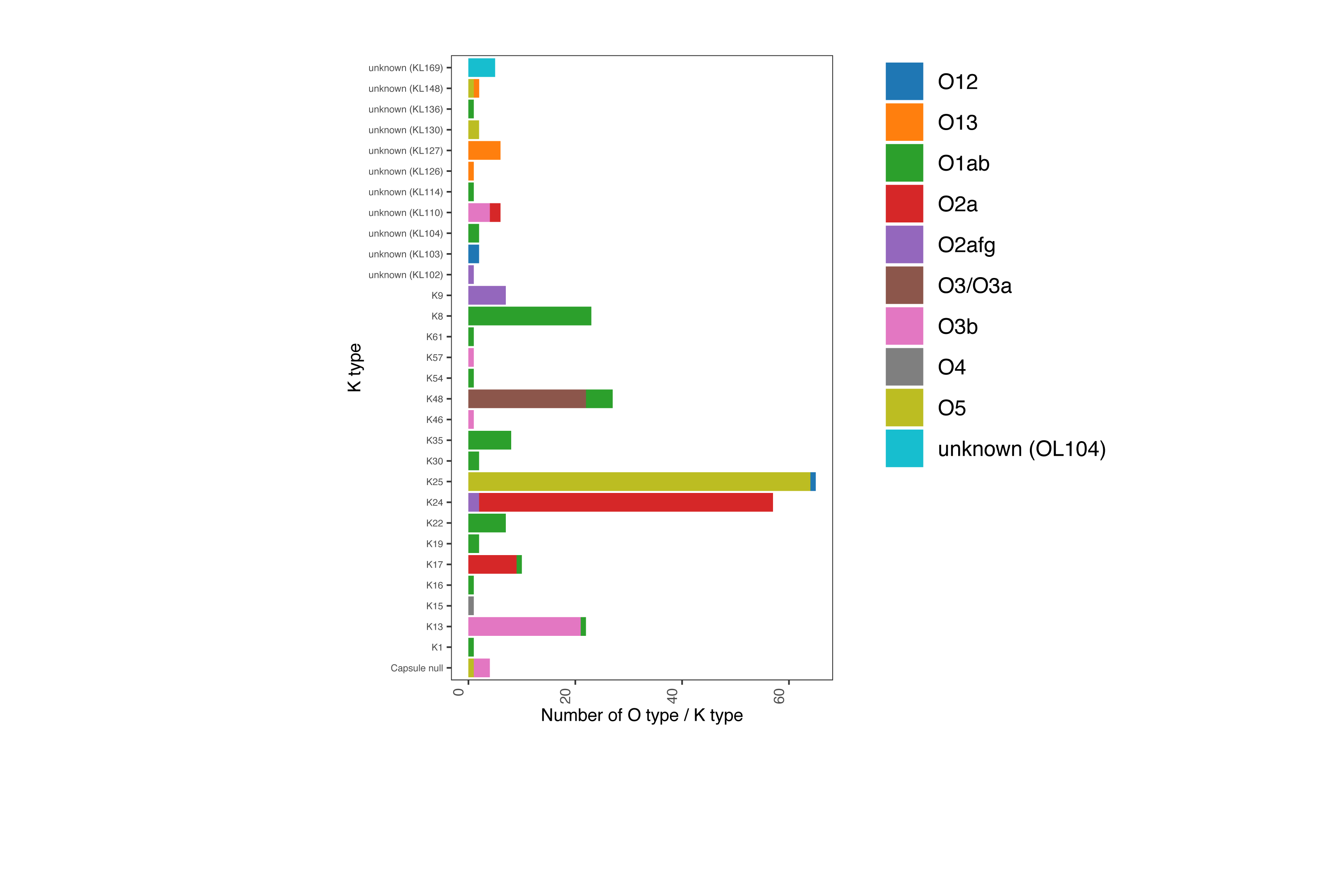

Supplement: S7 Fig — Each bar represents the number of isolates with a given capsular (K) type, colored by associated O antigen type. Dominant K types include K25, K24, K48, K13 and K8. (TIF) [file pgph.0006468.s009.tif]
