# Supplementary material for: Integrated epidemiologic investigation and genomic confirmation of a Klebsiella pneumoniae neonatal sepsis outbreak in Botswana
Source: PLOS Glob Public Health. 2026 Jun 15;6(6):e0006468. doi: 10.1371/journal.pgph.0006468 (PMC13268195; doi:10.1371/journal.pgph.0006468)
Supplement: Attachment — Submitted filename: Response to Reviewers.docx [file pgph.0006468.s011.docx]

**Dear Reviewers and Editors of PLOS Global Public Health,**

Thank you for your thorough and thoughtful review of our manuscript, "Integrated epidemiologic investigation and genomic confirmation of a Klebsiella pneumoniae neonatal sepsis outbreak in Botswana" (PGPH-D-26-00262). Below, we have responded to the reviewer’s comments, and we believe that our manuscript is improved based on their questions and recommendations. We thank the reviewers for their thoughtful and constructive feedback. We have addressed all comments and made revisions accordingly, as detailed below.

**Reviewer #1**

We appreciate the reviewer’s positive evaluation of the manuscript and their recognition of its methodological rigor and relevance to resource-constrained settings.

**Comment:**
*The manuscript requires a few minor, primarily grammatical, revisions.*

**Response:**
We have carefully reviewed the manuscript and made minor grammatical and stylistic edits throughout to improve clarity and readability.

**Reviewer #2**

We thank the reviewer for their thoughtful feedback and for recognizing the robustness and real-world impact of our work.

**Abstract**

**Comment:**
*Lines 50–52: Suggest breaking this into two sentences as initially it was a bit confusing to read given the length of this sentence.*

**Response:**
We agree and have revised this sentence by splitting it into two shorter sentences to improve clarity.

**Methods**

**Comment:**
*Provide more details regarding clinical indications for blood cultures.*

**Response:**
We have added clarification in lines 129-131 that blood cultures were obtained based on clinical signs and symptoms concerning for BSI, including standard neonatal sepsis indicators (e.g., temperature instability, respiratory distress, feeding intolerance, lethargy), in accordance with local clinical protocols.

**Comment:**
*Provide information on blood culture volume or target volume.*

**Response:**
We have added a statement in lines 130-131 describing the typical target blood culture volume in this setting (e.g., 1 mL per kg of infant bodyweight), acknowledging that collection volume may vary in practice due to clinical constraints in neonates.

**Comment:**
*Line 137: Capitalize “Gram-negative.”*

**Response:**
We appreciate the reviewer’s attention to detail. In line with standard scientific style conventions, “gram-negative” is not typically capitalized when used as a descriptor. We have therefore retained the lowercase usage and ensured that terminology is used consistently throughout the manuscript.

**Comment:**
*Genomic analysis: clarify timing and location (concurrent vs later; Botswana vs CHOP).*

**Response:**
We have clarified in lines 157-158 that genomic sequencing was performed retrospectively after the surveillance period. We have also specified the location where sequencing was conducted (USA).

**Results**

**Comment:**
*Line 214: Specify when the nine-week outbreak period occurred.*

**Response:**
We have revised the text in line 224 to specify when in the surveillance period (month 3) when the outbreak occurred. We have avoided using overt reference to specific calendar months when the outbreak occurred for sensitivity reasons.

**Comment:**
*Line 219: Clarify why MDR-Kpn isolates were deemed presumptive and consider moving explanatory text.*

**Response:**
We appreciate this suggestion. Isolate identification was considered “presumptive” if done using manual methods (which is prone to human error). In lines 115-117, we mention that “MDR-Kpn species were identified presumptively through visual growth on the selective media, and later confirmed by WGS.”

**Comment:**
*Lines 289–290: Remove redundant sentence regarding carbapenemase genes.*

**Response:**
We agree and have removed the redundant sentence.

**Comment:**
*Lines 299–303: Consider moving explanatory sentence to the Discussion.*

**Response:**
We agree that this text was more interpretive than descriptive. It has been moved to the Discussion section under limitations.

**Journal Requirements**

We have also addressed the editorial and formatting requirements outlined by the journal:

- A complete Financial Disclosure statement is available at the end of the manuscript which includes funding sources, author initials, and funder roles:
  - “This study was supported by the Gates Foundation grant (INV065400 to JS, SEC, KB and AMM) and the U.S. Centers for Disease Control & Prevention CDC-RFA-CK21-2104 Global Healthcare Detection and Response (NU3HCK000012 to JS, EL). PJP, KB and AMM report support from the Center for Microbial Medicine at CHOP. The funders had no role in study design, data collection and analysis, decision to publish, or preparation of the manuscript.”
- The Data Availability Statement has been updated to include repository details and direct access links where applicable.
- The manuscript file has been uploaded in the required format (.docx), and all figures and supplementary materials have been reformatted and submitted per journal guidelines.
- The reference list has been reviewed for completeness and accuracy.
