# Supplementary material for: Integrated epidemiologic investigation and genomic confirmation of a Klebsiella pneumoniae neonatal sepsis outbreak in Botswana
Source: PLOS Glob Public Health. 2026 Jun 15;6(6):e0006468. doi: 10.1371/journal.pgph.0006468 (PMC13268195; doi:10.1371/journal.pgph.0006468)
Supplement: Peer review history

# Author comment: Author response to Decision Letter 0

**DOI:** 10.1371/journal.pgph.0006468.r001

**Transfer Alert**

This paper was transferred from another journal. As a result, its full editorial history (including decision letters, peer reviews and author responses) may not be present.

22 Jan 2026

# Peer review: Decision Letter 0

**DOI:** 10.1371/journal.pgph.0006468.r002

**Authors:** Ioana D. Olaru

14 Apr 2026

PGPH-D-26-00262

Integrated epidemiologic investigation and genomic confirmation of a Klebsiella pneumoniae neonatal sepsis outbreak in Botswana

PLOS Global Public Health

Dear Dr. Moustafa,

Thank you for submitting your manuscript to PLOS Global Public Health. After careful consideration, we feel that it has merit but does not fully meet PLOS Global Public Health’s publication criteria as it currently stands. Therefore, we invite you to submit a revised version of the manuscript that addresses the points raised during the review process.

Two reviewers have evaluated your manuscript and provided their comments below.

Please submit your revised manuscript by May 14 2026 11:59PM. If you will need more time than this to complete your revisions, please reply to this message or contact the journal office at globalpubhealth@plos.org. When you're ready to submit your revision, log on to https://www.editorialmanager.com/pgph/ and select the 'Submissions Needing Revision' folder to locate your manuscript file.

Please include the following items when submitting your revised manuscript:

- A letter that responds to each point raised by the editor and reviewer(s). You should upload this letter as a separate file labeled 'Response to Reviewers'.
- A marked-up copy of your manuscript that highlights changes made to the original version. You should upload this as a separate file labeled 'Revised Manuscript with Track Changes'.
- An unmarked version of your revised paper without tracked changes. You should upload this as a separate file labeled 'Manuscript'.

Guidelines for resubmitting your figure files are available below the reviewer comments at the end of this letter.

As the corresponding author, your ORCID iD is verified in the submission system and will appear in the published article. PLOS supports the use of ORCID, and we encourage all coauthors to register for an ORCID iD and use it as well. Please encourage your coauthors to verify their ORCID iD within the submission system before final acceptance, as unverified ORCID iDs will not appear in the published article. *Only*  the individual author can complete the verification step; PLOS staff *cannot*  verify ORCID iDs on behalf of authors.

We look forward to receiving your revised manuscript.

Kind regards,

Ioana D. Olaru

Academic Editor

PLOS Global Public Health

Journal Requirements:

1. Please provide a detailed online Financial Disclosure statement. This is published with the article. It must therefore be completed in full sentences and contain the exact wording you wish to be published.

a) State the initials, alongside each funding source, of each author to receive each grant. For example: “This work was supported by the National Institutes of Health (####### to AM; ###### to CJ) and the National Science Foundation (###### to AM).”

b) State what role the funders took in the study. If the funders had no role in your study, please state: “The funders had no role in study design, data collection and analysis, decision to publish, or preparation of the manuscript.”

For more information, please go to our submission guidelines:

https://journals.plos.org/globalpublichealth/s/submission-guidelines#loc-financial-disclosure-statement

2. Please ensure that the funders and grant numbers match between the Financial Disclosure field and the Funding Information tab in your submission form. Note that the funders must be provided in the same order in both places as well.

3. Please note that your Data Availability Statement is currently missing the repository name and a direct link to access each database. If your manuscript is accepted for publication, you will be asked to provide these details on a very short timeline. We therefore suggest that you provide this information now, though we will not hold up the peer review process if you are unable.

4. Please upload your main article file as a .doc, .docx or .rtf file.

5. Please provide separate main figure files in .tif or .eps format only and ensure that all files are under our size limit of 10MB.

For more information about how to convert your figure files please see our guidelines: https://journals.plos.org/globalpublichealth/s/figures

6. We notice that your supplementary figures are included in the manuscript file. Please remove them and upload them with the file type 'Supporting Information'. Please ensure that each Supporting Information file has a legend listed in the manuscript before or after the references list.

7. If the reviewer comments include a recommendation to cite specific previously published works, please review and evaluate these publications to determine whether they are relevant and should be cited. There is no requirement to cite these works unless the editor has indicated otherwise.

8. Please review your reference list to ensure that it is complete and correct. If you have cited papers that have been retracted, please include the rationale for doing so in the manuscript text, or remove these references and replace them with relevant current references. Any changes to the reference list should be mentioned in the rebuttal letter that accompanies your revised manuscript. If you need to cite a retracted article, indicate the article’s retracted status in the References list and also include a citation and full reference for the retraction notice.

[Note: HTML markup is below. Please do not edit.]

Reviewers' comments:

Reviewer's Responses to Questions

**Comments to the Author**

1. Does this manuscript meet PLOS Global Public Health’s publication criteria? Is the manuscript technically sound, and do the data support the conclusions? The manuscript must describe methodologically and ethically rigorous research with conclusions that are appropriately drawn based on the data presented.

Reviewer #1: Yes

Reviewer #2: Yes

**********

2. Has the statistical analysis been performed appropriately and rigorously?

Reviewer #1: Yes

Reviewer #2: Yes

**********

3. Have the authors made all data underlying the findings in their manuscript fully available (please refer to the Data Availability Statement at the start of the manuscript PDF file)?

The PLOS Data policy requires authors to make all data underlying the findings described in their manuscript fully available without restriction, with rare exception. The data should be provided as part of the manuscript or its supporting information, or deposited to a public repository. For example, in addition to summary statistics, the data points behind means, medians and variance measures should be available. If there are restrictions on publicly sharing data—e.g. participant privacy or use of data from a third party—those must be specified.

Reviewer #1: Yes

Reviewer #2: Yes

**********

4. Is the manuscript presented in an intelligible fashion and written in standard English?

PLOS Global Public Health does not copyedit accepted manuscripts, so the language in submitted articles must be clear, correct, and unambiguous. Any typographical or grammatical errors should be corrected at revision, so please note any specific errors here.

Reviewer #1: Yes

Reviewer #2: Yes

**********

5. Review Comments to the Author

Please use the space provided to explain your answers to the questions above. You may also include additional comments for the author, including concerns about dual publication, research ethics, or publication ethics. (Please upload your review as an attachment if it exceeds 20,000 characters)

Reviewer #1: The manuscript meets publication criteria. It is well-written. It successfully identifies gaps in prevention, diagnosis, and management of neonatal sepsis in resource-constrained areas. The methodology is sound. The data supports the conclusions. The authors describe successful implementation of multi-modal diagnostics to improve the management of an infectious epidemic in a resource-constrained setting. They acknowledge that the equipment used in the study may still be financially out of reach for many resource-constrained settings, which is an important limitation. The manuscript requires a few minor, primarily grammatical, revisions.

Reviewer #2: The authors describe an epidemiological surveillance and outbreak study of K. pneumoniae in a neonatal unit located in Botswana. Their work is robust and has real world applications, including outbreak management during the study itself. I commend them for their excellent work and the manuscript is very clearly written. Their data are an important addition to the field and the figures/tables (both in the paper and supplemental) are well done.

I have a few minor suggestions and questions for their consideration.

Abstract:

- Lines 50-52: Suggest breaking this into two sentences as initially it was bit confusing to read given the length of this sentence.

Methods:

- In the section on blood culture isolates, is it possible to provide more details regarding what the clinical indications were? Presumably they were for signs/symptoms of BSI?

- It would also be helpful to know what volume of blood culture were drawn, or what is generally targeted as the goal volume.

- Line 137, capitalize G in Gram-negative BSI

- Genomic analysis: was this done concurrent to the microbiologic work up, or later on? Additionally, was the sequencing done in Botswana or at CHOP? As the authors acknowledge in the discussion, WGS technology is not readily available in many LMICs. Further detail of when and where WGS was done would add some additional context.

Results:

-Line 214: The authors gave the duration of the study in the methods as November 2022-November 2023 and eluded to the outbreak occurring in the first three months of the study. It may be helpful to state when the nine-week period occurred by month.

-Line 219: Initially I was curious as to why the MDR-Kpn species were deemed as presumptive, but I believe this is answered later on in Lines 235-237 with additional detail about the ESBL media. The authors could move the later lines 235-237 to the discussion as it is somewhat explanatory of the results, or move it up to the information presented in line 219 if this is indeed why the species were presumed to be MDR-Kpn.

- Line 289-290: Could remove the sentence regarding carbapenemase genes as this is stated again in the concluding sentence of this paragraph.

- Lines 299-303: Consider moving this sentence to the discussion and it reads as explanatory rather than results.

**********

6. PLOS authors have the option to publish the peer review history of their article (what does this mean?). If published, this will include your full peer review and any attached files.

**Do you want your identity to be public for this peer review?**  If you choose “no”, your identity will remain anonymous but your review may still be made public.

For information about this choice, including consent withdrawal, please see our Privacy Policy.

Reviewer #1: No

Reviewer #2: No

**********

[NOTE: If reviewer comments were submitted as an attachment file, they will be attached to this email and accessible via the submission site. Please log into your account, locate the manuscript record, and check for the action link "View Attachments". If this link does not appear, there are no attachment files.]

Figure Resubmissions:

While revising your submission, we strongly recommend that you use PLOS’s NAAS tool (https://ngplosjournals.pagemajik.ai/artanalysis) to test your figure files. NAAS can convert your figure files to the TIFF file type and meet basic requirements (such as print size, resolution), or provide you with a report on issues that do not meet our requirements and that NAAS cannot fix.

After uploading your figures to PLOS’s NAAS tool - https://ngplosjournals.pagemajik.ai/artanalysis, NAAS will process the files provided and display the results in the "Uploaded Files" section of the page as the processing is complete. If the uploaded figures meet our requirements (or NAAS is able to fix the files to meet our requirements), the figure will be marked as "fixed" above. If NAAS is unable to fix the files, a red "failed" label will appear above. When NAAS has confirmed that the figure files meet our requirements, please download the file via the download option, and include these NAAS processed figure files when submitting your revised manuscript.

# Author comment: Author response to Decision Letter 1

**DOI:** 10.1371/journal.pgph.0006468.r003

23 Apr 2026

Attachment Submitted filename: Response to Reviewers.docx

# Editor report: Decision Letter 1

**DOI:** 10.1371/journal.pgph.0006468.r004

**Authors:** Ioana D. Olaru

28 Apr 2026

Integrated epidemiologic investigation and genomic confirmation of a Klebsiella pneumoniae neonatal sepsis outbreak in Botswana

PGPH-D-26-00262R1

Dear Dr. Moustafa,

We are pleased to inform you that your manuscript 'Integrated epidemiologic investigation and genomic confirmation of a Klebsiella pneumoniae neonatal sepsis outbreak in Botswana' has been provisionally accepted for publication in PLOS Global Public Health.

Before your manuscript can be formally accepted you will need to complete some formatting changes, which you will receive in a follow up email. A member of our team will be in touch with a set of requests.

Please note that your manuscript will not be scheduled for publication until you have made the required changes, so a swift response is appreciated.

IMPORTANT: The editorial review process is now complete. PLOS will only permit corrections to spelling, formatting or significant scientific errors from this point onwards. Requests for major changes, or any which affect the scientific understanding of your work, will cause delays to the publication date of your manuscript.

If your institution or institutions have a press office, please notify them about your upcoming paper to help maximize its impact. If they'll be preparing press materials, please inform our press team as soon as possible -- no later than 48 hours after receiving the formal acceptance. Your manuscript will remain under strict press embargo until 2 pm Eastern Time on the date of publication. For more information, please contact globalpubhealth@plos.org.

Thank you again for supporting Open Access publishing; we are looking forward to publishing your work in PLOS Global Public Health.

Best regards,

Ioana D. Olaru

Academic Editor

PLOS Global Public Health

***********************************************************

Reviewer Comments (if any, and for reference):
